# Preventive Medicine in General Practice

**Published:** 1988-11

**Authors:** Jonathan Williams

**Affiliations:** General Practice Vocational Trainee, Bristol

## Abstract

Ten final year medical students observed 360 consultations undertaken by 10 West Country General Practitioners. In 31% of the consultations, General Practitioners initiated preventive medical care. These consultations lasted 1.5 minutes longer than those in which no preventive measures were initiated, and most patients reacted with interest or enthusiasm to the preventive care. In 11% of the consultations patients themselves sought preventive advice. These consultations were not significantly longer than consultations in which patients sought only more immediate help or symptom relief. General Practitioners reacted favourably to 85% of the preventive enquiries.


### Preventive Medicine in General Practice: An Evaluation of the Consultations of Ten General Practitioners

**Jonathan Williams, M.B., Ch.B. D.C.H.**
General Practice Vocational Trainee, Bristol

### SUMMARY
Ten final year medical students observed 360 consultations undertaken by 10 West Country General Practitioners. In 31% of the consultations, General Practitioners initiated preventive medical care. These consultations lasted 1.5 minutes longer than those in which no preventive measures were initiated, and most patients reacted with interest or enthusiasm to the preventive care. In 11% of the consultations patients themselves sought preventive advice. These consultations were not significantly longer than consultations in which patients sought only more immediate help or symptom relief. General Practitioners reacted favourably to 85% of the preventive enquiries.

### INTRODUCTION
Preventive medicine is not new. In 1919, the Chief Medical Officer stated; "The first duty of medicine is not to cure disease, but to prevent it" (1). However, although prevention has been an important feature of twentieth century medical practice, most resources have been channelled towards curative medicine (2)–(4). Nevertheless, awareness of the importance of the prevention of diseases and the promotion of health in medical practice has increased rapidly in recent years (5)–(8). General Practice has been identified as an ideal setting for prevention (9), (10); the general practitioner has frequent contact with the majority of a defined population, including those at risk. He or she is perceived as credible and trustworthy, communicates on a one to one basis with the patient and is supported by a primary care team. The potential for preventive medicine in general practice is beginning to be explored (11)–(15).

Despite recent advances, the Royal College of General Practitioners in 1981 (16) professed ignorance of the extent and thoroughness with which General Practitioners undertook preventive care. A subsequent random selection of 5% of 1302 tape recorded consultations undertaken by 16 General Practitioners showed that prevention was discussed in 23% of consultations (17). A postal questionnaire of 193 General Practitioners (18) found a high level of interest in certain preventive activities, particularly smoking prevention. However, an evaluation of 8,500 patients, notes from 38 practices (19) found that information about smoking habits was recorded in only 23% of the notes.

Many further questions remain. "We need to ask how many patients on a practice list will visit their doctors when well in order to discuss preventive measures such as diet, exercise, or means of changing a health-threatening habit, and how much time or informed discussion general practitioners will be willing to devote to the interview" (Taylor, 1982). A recent survey of patients from 47 practices (20) suggested that patients are concerned about their lifestyles and would welcome relevant advice, but in many cases they felt that they received insufficient health education from their General Practitioners. On the other hand, Smail (1982) warns of the ".... dangers in an over-enthusiastic approach to preventive care, particularly if the doctor gives a great deal of heavy-handed proscriptive advice. There is some risk of alienating the patient ...." This study was designed to record the quantity and nature of preventive care undertaken during consultations of ten West Country general practitioners. The reaction of the general practitioner to enquiries from the patient, and the response and attitudes of patients to the preventive advice during the consultation was also assessed, as was the duration of each consultation.

### METHOD
Final year medical students from the University of Bristol spend two weeks attending surgeries and observing consultations of general practitioners in the South Western Region. Of forty general practitioners due to receive a student during the same two week period, ten, whose practices were in market towns of comparable size and socio-economic environment in Devon and Somerset, were selected for study. A pilot study of forty consultations was undertaken to ensure the feasibility of recording the necessary information, without disturbing the patient or the doctor during the consultation. The protocol was sent to the ten medical students who obtained the permission of their general practitioners to record certain information about each consultation; viz., the age and 'social class' of each patient (divided into three classes, 'professional', 'clerical' or 'manual'). However, in order to avoid the possibility of altering the general practitioner's normal behaviour during consultation, the nature of the other information recorded was not disclosed.

The protocol defined prevention: "the promotion of a healthy lifestyle", and primary or secondary prevention: "the avoidance of the causes of any disease, or its diagnosis and treatment at an early preclinical stage". Examples of preventive medical care given were: stopping smoking, measuring blood pressure, combating obesity by diet or exercise, screening procedures, family planning, accident avoidance, stress avoidance, immunisation and control of alcohol intake.

All prevention initiated by patients was noted and thegeneral practitioner's reaction to the preventive enquiry was graded (a) enthusiastic (b) interested (c) disinterested or (d) dismissive. Preventive action or advice initiated by the general practitioner, either related to a problem presented by the patient (for instance, discussion about smoking with a patient presenting with a cough) or unrelated (for example, measuring the blood pressure of a patient with a sprained ankle) was recorded. The reaction of the patient during the consultation to the doctor's preventive measure was also graded as above. Every consultation was timed to the nearest minute.

The study was undertaken in the second week of the student attachment when the general practitioner had become accustomed to the presence of an observer. Students were asked to begin at the start of a day's surgery, and observe as many consecutive consultations undertaken by the general practitioner as possible in the course of routine surgeries; thus special surgeries such as ante-natal or child clinics were not included. Information about each consultation was recorded on a questionnaire.

### RESULTS

All ten general practitioners agreed to participate in the study, and 360 consultations were observed (mean=36 consultations, range 15–112 consultations). Amongst these consultations, 16% involved patients under fifteen years, 34% of patients were aged fifteen to forty four, 28% were forty five to sixty four, and 22% were sixty five years and above. Fifty four per cent of patients consulting were female, 17% of patients were 'professional', 55% were 'clerical' and 28% were of 'manual' social class.

In the 360 consultations, 39 patients (10.8%) enquired about prevention; three of these patients asked two questions on prevention making a total of 42 patient-initiated preventive enquiries. These enquiries are shown in Table I.

**Table I**

**Type and Frequency of Patient-Initiated Prevention**

| *Patient-Initiated Prevention* | *Number* |
|---|---|
| Enquiry about blood pressure | 12 |
| Advice re weight, diet or exercise | 9 |
| Family planning | 7 |
| Request for a screening procedure | 5 |
| Advice re smoking | 3 |
| Lifestyle/stress | 2 |
| Immunisation | 2 |
| Accident avoidance | 1 |
| Alcohol | 1 |
| Total | 42 |

The patient-initiated prevention was increasingly sought from manual through to professional classes, although this trend did not reach statistical significance. Patients aged 14 and under asked fewer questions on prevention than older patients, and females asked more questions about prevention (not statistically significant). General practitioners reacted favourably to the majority of enquiries made by patients about prevention (see Table II).

**Table II**

**Reaction of General Practitioners to Patient-Initiated Prevention**

| *Reaction* | *Number* | *% of Consultations involving Patient-Initiated Prevention* |
|---|---|---|
| Enthusiastic | 17 | 43.6 |
| Interested | 16 | 41.0 |
| Disinterested | 4 | 10.3 |
| Dismissive | 1 | 10.3 |
| Dismissive | 1 | 2.6 |
| Not recorded | 1 | 2.6 |
| Not applicable | 321 | |
| Total | 360 | |

The general practitioners initiated 149 preventive measures in the course of 111 (30.8%) of the 360 consultations. (Table III) 68% of the preventive care given was related to the patient's presenting complaint.

**Table III**

**Type and Frequency of G.P.-Initiated Prevention**

| *Preventive Measure* | *Number of Patients* | *% of Total Consultations* |
|---|---|---|
| Smoking reduction | 49 | 13.6 |
| B.P. measure | 42 | 11.7 |
| Weight/diet/exercise | 22 | 6.1 |
| Accident avoidance | 13 | 3.6 |
| Lifestyle/stress | 10 | 2.8 |
| Screening | 8 | 2.2 |
| Alcohol | 3 | 0.8 |
| Immunisation | 1 | 0.3 |
| Family planning | 1 | 0.3 |
| Total | 149 | |

There was no significant difference in the preventive care received by males and females, or between social classes. However, as shown in Table IV, children under fifteen years received significantly less preventive care from their general practitioners than older patients.

**Table IV**

**G.P.-Initiated Prevention By Age**

| *Age (years)* | *Prevention* | *No Prevention* | *Total Consultations* | *% Prevention by Age Group* |
|---|---|---|---|---|
| 0–14 | 6 | 50 | 56 | 10.7 |
| 15–44 | 46 | 78 | 124 | 37.1 |
| 45–64 | 30 | 71 | 101 | 29.7 |
| 65+ | 29 | 50 | 79 | 36.7 |
| Total | 111 | 49 | 360 | |

$\chi 2$=14.2 deg. of freedom =3 P<0.01

The proportion of consultations in which preventive care was given, varied considerably between the ten general practitioners (range 6.6%–86.7%, mean=32%). The majority of patients reacted favourably at the time of the consultation to the preventive medical care (Table V). The reaction of patients receiving preventive advice which could be considered inappropriate (for instance an enquiry about smoking in non-smokers) was recorded as such.

**Table V**

**Reaction of Patients to G.P.-Initiated Prevention**

| *Reaction* | *Patient* | *% of Consultations Involving G.P.-Initiated Prevention* |
|---|---|---|
| Enthusiastic | 20 | 18.0 |
| Interested | 42 | 37.8 |
| Disinterested | 16 | 14.4 |
| Dismissive | 4 | 3.6 |
| Not appropriate | 29 | 26.1 |
| Total | 111 | |

The average length of the 360 consultations was 7 minutes 25 seconds (S.D.=4 mins 25 secs). The mean duration of consultations in which the patient initiated prevention (n=39) was 8 mins 5 secs long (S.D.=4 mins 25 secs), and not significantly different to the mean duration of consultations in which no prevention was initiated by the patient (n=321, mean=7 min 20 secs, S.D.=4 mins 25 secs). However, consultations in which the general practitioner initiated prevention (n=111) were statistically significantly longer (mean=8 mins 25 secs, S.D.=4 mins 30 secs) than the consultations (n=249) during which the general practitioner did not offer preventive medical care (mean=6 min 55 secs. S.F.=4 mins 15 secs P<0.01).

### DISCUSSION

The number of consultations of each general practitioner studied differed widely, because students spent varying amounts of time with other members of the primary health care team during their practice attachments. The consultation behaviour of the ten general practitioners who participated in this study is not necessarily representative of all general practitioners and could have been altered by the presence of a student observer. The study recorded only a single consultation between a patient and his/her general practitioner, and therefore does not take into account preventive care which may have arisen during previous meetings. Furthermore, the age, sex and class structure of the population in market towns in Somerset and Devon, the level of morbidity, and the patients' expectations of primary health care could also differ in other practices. Nevertheless, the findings have implications for approaches to preventive care in general practice.

Sixty-eight per cent of the consultations in which the general practitioner initiated prevention were related to the patient's presenting problems. Fowler, (1984) suggested that patients may be better motivated to accept preventive advice if they are concerned about possible disease. The HESU study (1982) suggested that many General Practitioners did not explore patients' pre-existing knowledge or beliefs about health issues, and this reduced the educational value of the consultation. However, when patients took a more active role in the consultation, they were more likely to understand, and therefore comply with advice and treatment given by their doctor.

General practitioners were observed to offer significantly less preventive care to children under fifteen. Some of this age group included very young children, for whom certain preventive measures such as direct health education, would have been inappropriate. In other instances, general practitioners may have felt that the responsibility for preventive care lay with the parents. Nevertheless, it would seem sensible to aim health education programmes at children, as their behaviour patterns with regard to smoking, diet and exercise are less likely to be permanently established than later in life.

Consultations in which the General Practitioners initiated preventive care lasted 1.5 minutes longer than those in which no preventive care was given. However, there is evidence that this extra time spent on preventive education is worthwhile. For example, it has been shown that patients' smoking habits can be modified by preventive education (21). It has also been suggested that greater discussion of health issues during consultation may reduce repeat consultations by patients, thereby saving time in the longer term (17).

The Royal College of General Practitioners (1981) suggested that "Anticipatory care"—the alliance of prevention, care and cure— ". . . . should be the main direction of growth for primary medical services". Forty per cent of the consultations reviewed in this study included preventive measures, and both patients and doctors had positive attitudes towards them. However, prevention initiated by General Practitioners lengthened the consultations. Further study should consider the cost-effectiveness of preventive care.

### REFERENCES

1. NEWMAN, G. (1919) An outline of the practice of preventive medicine. London, H.M.S.O.
2. BRITISH MEDICAL ASSOCIATION (1946) A Charter for Health.
3. OWEN, D. (1976) In sickness and in health. London, Quartet Books.
4. KENNEDY, I. (1981) The Unmasking of Medicine. London, Allen and Unwin.
5. ROYAL COLLEGE OF PHYSICIANS (1962) Smoking and Health. London, Pitman Medical.
6. DEPARTMENT OF HEALTH AND SOCIAL SECURITY (1976) Prevention and health: everbody's business.
7. WORLD HEALTH ORGANISATION (1978) Alma-Ata: Primary Health Care, Report of the international conference.
8. DOLL, R. (1983) Prospects for prevention. Br. Med. J. **286**, 445–453.
9. ZANDOR, L.I. (1982) Making a start. Br. Med. J. **284**, 1241–1242.
10. FOWLER, G. (1984) The challenge of prevention. The Practitioner, **228**, 1143–1147.
11. STOTT, H.C.H. and DAVIS, R.M. (1979) The exceptional potential in each primary care consultation. Journal of the Royal College of General Practitioners **29**, 201–205.
12. TAYLOR, K.B. (1982). Preventive medicine in General Practice. *Br. Med. J.* **284**, 921–922.
13. SMAIL, S.A. (1982) Opportunities for prevention: the consultation. *Br. Med. J.* **284**, 1092–1093.
14. GRAY, M. and FOWLER, G. (1983) Preventive Medicine in General Practice. Oxford, Oxford University Press.
15. FULLARD, E., FOWLER, G., and GRAY, M. (1984). Facilitating prevention in primary care. *Br. Med. J.* **289**, 1585–7.
16. ROYAL COLLEGE OF GENERAL PRACTITIONERS (1981) Health and Prevention in Primary Care. Reports From General Practice No. 18.
17. HEALTH EDUCATION STUDIES UNIT (1982) The Patient Project. Health Education Council, London.
18. CATFORD, J.C. and NUTBEAM, D. (1984) Prevention in Practice: What Wessex General Practitioners are doing. *Br. Med. J.* **288**, 832–834.
19. FLEMING, D.M. and LAWRENCE, M.S.T.A. (1981) An evaluation of recorded information about preventive measures in thirty eight practices. Journal of the Royal College of General Practitioners, *31*, 615–620.
20. WALLACE, P.G., BRENNAN, P.J., HAINES, A.P. (1987). Are general practitioners doing enough to promote healthy lifestyle? Findings of the Medical Research Council's general practice research framework study on life and health. *Br. Med. J.* **294**, 940–942.
21. RUSSELL, M.A.H., WILSON, C., TAYLOR, C. *et al* (1979) Effects of General Practitioners' advice against smoking. *Br. Med. J.* **(ii)**, 231–235.

### ACKNOWLEDGEMENTS

I am most grateful to the ten general practitioners and medical students who agreed to participate in the study, and to Dr Robin Philipp, Mr Tony Hughes and Mrs Kathleen Little of the Department of Epidemiology and Community Medicine for their generous support.

### ADDRESS FOR CORRESPONDENCE

Dr. J.D. Williams

11 Upton Road

Southville

Bristol BS3

